# Intact delay discounting but more optimal reward‐based decisions in anorexia nervosa during an experiential task

**DOI:** 10.1111/pcn.70041

**Published:** 2026-02-18

**Authors:** Fabio Bernardoni, Joseph A. King, Maria Seidel, Martin Schoemann, Nina Kasaeva, Katrin Gramatke, Kerstin Weidner, Veit Roessner, Stefan Scherbaum, Stefan Ehrlich

**Affiliations:** ^1^ Translational Developmental Neuroscience Section, Division of Psychological and Social Medicine and Developmental Neurosciences, Faculty of Medicine Technische Universität Dresden Dresden Germany; ^2^ Department of Psychology, Faculty of Natural sciences Technische Universität Dresden Dresden Germany; ^3^ Department of Child and Adolescent Psychiatry, Faculty of Medicine, University Hospital C. G. Carus Technische Universität Dresden Dresden Germany; ^4^ Department of Psychotherapy and Psychosomatic Medicine, Faculty of Medicine, University Hospital C. G. Carus Technische Universität Dresden Dresden Germany; ^5^ Department of Child and Adolescent Psychiatry, Faculty of Medicine, University Hospital C. G. Carus Technische Universität Dresden, German Center for Child and Adolescent Health (DZKJ) Dresden Germany; ^6^ Eating Disorder Treatment and Research Center, Department of Child and Adolescent Psychiatry, Faculty of Medicine Technische Universität Dresden Dresden Germany

**Keywords:** anorexia nervosa, decision‐making, delay discounting, experiential task, self‐control

## Abstract

**Aims:**

Individuals with anorexia nervosa (AN) have an apparent increased capacity to delay gratification and forgo immediate food rewards in their long‐term pursuit of thinness. Prior research probed this capacity using delay discounting tasks that assess how rapidly the subjective value of rewards decreases as a function of time until receipt. However, significant effects in AN were mostly subtle or absent using traditional tasks. Here, we tested whether altered delay discounting might be revealed in AN during an experiential gamified task.

**Methods:**

Eighty acutely underweight females with AN and pairwise age‐matched female healthy controls (HC) made binary choices moving an avatar to collect rewards of varying magnitudes and distances. The presence of an overall time limit rendered one choice optimal (i.e. higher cost–benefit ratio) in each trial. We tested group differences in delay/distance discounting and used signal detection theory to determine whether decision patterns were due to inherent preferences (e.g. for immediate rewards) or acuity to detect optimal choices.

**Results:**

No group differences in delay discounting parameters were found, but participants with AN favored smaller, closer rewards when this was the optimal strategy. This was neither related to an *a priori* bias toward immediate/delayed rewards nor enhanced choice acuity.

**Conclusion:**

Even in a more realistic experiential task, individuals with AN did not exhibit reduced delay discounting. Instead, because choosing the closer option more frequently required more decisions overall, our findings may reflect increased task engagement and align with the notion of greater willingness to exert effort for reward in AN.

Anorexia nervosa (AN) is a severe psychiatric disorder characterized by persistent food restriction, intense fear of weight gain, and a distorted body image, often leading to life‐threatening underweight.^1^ Its hallmark features – such as extreme dietary control and the ability to forgo immediate rewards – suggest alterations in value‐based decision‐making and temporal discounting, processes that may offer insight into the mechanisms underlying the disorder.^2^, ^3^ The unusually high capacity of individuals with AN to resist hunger and forgo eating has been hypothesized to reflect one facet of heightened self‐control and/or altered reward processing.^2^, ^4^, ^5^ This willingness to wait longer for larger rewards is evident in behaviors such as restricting food intake, engaging in excessive exercise, tolerating extreme cold,^6^ and maintaining high levels of academic performance.^7^ A growing number of studies have employed intertemporal choice (or delay discounting [DD]) tasks to quantify the excessive preference for delayed rewards often observed in individuals with AN.^8^ In DD tasks, participants choose between a smaller, immediate reward (‘smaller‐sooner’, SS) and a larger, delayed reward (‘larger‐later’, LL), allowing for the measurement of the rate at which the subjective value of a reward decreases with delay. While steeper discounting curves have been reliably observed in individuals with deficient self‐control,^9^ particularly addiction,^10^ obesity,^11^ and Attention‐Deficit/Hyperactivity Disorder,^12^ comparatively few studies have found evidence of shallower discounting curves in AN.^13^, ^14^ In contrast, several studies found no differences or only AN‐subtype‐specific effects.^9^, ^15^, ^16^, ^17^, ^18^, ^19^, ^20^ However, individuals with AN have been found to self‐report a higher tendency to delay gratifications.^21^

One possible explanation for why so few experimental studies have found an effect is that these tasks ask the participant to carry out consecutive mostly theoretical choices, that is, without actually implementing a waiting period and without delivering the delayed reward during task execution (often one chosen reward was randomly selected as a real payoff). Such setups may not reflect realistic conditions and may not truly engage participants in making real choices.^22^, ^23^, ^24^ Delay discounting has been identified by the National Institute of Mental Health as a potential treatment target^25^ to nudge people toward making more advantageous choices^8^ and a quantitative estimation of it might allow clinicians to follow treatment progress in patients. In this study, we attempted to overcome the limitations of DD tasks previously employed in behavioral research in AN by incorporating real‐world costs and contextual factors, enabling a more ecologically valid assessment of decision‐making.^24^, ^26^, ^27^ To this end, we introduced a gamified paradigm, previously demonstrated in separate studies to dissociate between individuals with heroin addiction and specific phobias relative to HC,^28^, ^29^ where in each trial participants had to choose between moving an avatar toward a smaller, but closer reward or a larger, but more distant one. Importantly, after making a decision, participants had to wait until the avatar autonomously collected the reward (represented as a coin with value proportional to its size) before proceeding to the next trial, so the closer (more distant) option was also received sooner (later). Therefore, participants had to endure the cost of choosing delayed (distant) rewards, as opposed to most traditional tasks with merely abstract choices. At the end of each trial, the reward was added to a cumulative loot (which participants actually received as bonus compensation), making each choice relevant for the overall gain. It is also worth noting that while steep discounting has been associated with impulsivity and maladaptive behaviors such as drug and gambling addictions,^10^ DD can also be rationalized in terms of the opportunity costs of waiting. In the current experiential paradigm, choosing the LL option also resulted in a delay of the subsequent trial and, consequently, the chance to obtain another reward. Given that intertemporal choices can be profoundly influenced by contextual factors such as how alternative options are framed and subjective/state factors of the decision‐maker,^30^ it would be advantageous to study discounting rates in situations in which there is a ‘level playing field’ for all participants and an objectively optimal option. To this end, we implemented an overall time limit in our gamified DD task so that in each trial one option always promised greater monetary gain per unit of time. Depending on magnitude and delay, either the SS or LL reward could be optimal, and importantly, there was a substantial number of both SS‐optimal and LL‐optimal trials. Consequently, our paradigm allowed testing not only differences in discounting rates but also whether participants preferred the SS (or LL) reward despite its suboptimality. For example, a study using this paradigm clarified that increased discounting in heroin addiction reflects not only a heightened bias toward immediate gratification but also a reduced ability to choose the better option.^28^ Another study showed that individuals with phobia make less optimal choices in the presence of fear‐related stimuli.^29^

Based on the theory of overcontrol and excessive goal pursuit in AN,^31^ we expected that in our gamified task with an arguably more naturalistic design, we would (despite previous null findings using traditional intertemporal choice tasks) capture less discounting in this patient population. Furthermore, given that our task allowed us to differentiate objectively optimal (greater monetary gain per unit of time) from suboptimal decisions, our second aim was to test whether decision‐making in individuals with AN was more or less goal‐directed.

## Methods

### Sample description

Between August 2017 and January 2022, a total of 205 female volunteers between 12 and 26 years of age participated in the study: 80 acutely underweight patients with AN according to DSM‐5 (acAN, age range: 12.2–26.0 years) and 125 HC (age range: 12.2–25.9 years). The Institutional Review Board of the Technische Universität Dresden approved the study, and all participants (or their legal guardians) gave written informed consent. The sample size was much larger than in a prior study by Scherbaum *et al*.,^28^ which detected a group difference by employing 25 participants per group using a similar paradigm in individuals with heroin addiction. This sample had 93% power to detect a medium effect (Cohen's *d* = 0.5), 60% power to detect an effect as large as reported in a meta‐analysis by Amlung *et al*.^9^ (Cohen's *d* = 0.3), but only 35% power to detect a small effect (Cohen's *d* = 0.2).

To ascertain the absence or presence of a current eating disorder, the expert form of the Structured Interview for Anorexia and Bulimia Nervosa for DSM‐IV (SIAB‐EX)^32^ was conducted with all participants. Interviews were adapted to DSM‐5 criteria and carried out by clinically experienced and trained research assistants under the supervision of the attending child and adolescent psychiatrist with >15 years of clinical experience. acAN participants were admitted to specialized eating disorder treatment programs at the University Hospital Carl Gustav Carus Dresden and assessed within 96 h of beginning nutritional rehabilitation. Inclusion criteria for acAN were a body mass index (BMI) derived from objective measurement of height and weight below the 10th age percentile (if younger than 18 years) or 17.5 kg/m^2^ (if older than 18 years) and no recent weight gain. To be included in the HC group, participants had to have a BMI in the 10th–94th percentile range (if younger than 18 years) or in the range 18.5–28 kg/m^2^ (if older than 18 years), be eumenorrhoeic, and have no history of psychiatric illness or substance abuse, as assessed with a brief structured clinical interview^33^, ^34^ according to DSM‐IV. Participants of both study groups had no lifetime history of organic brain syndrome, schizophrenia, psychosis not otherwise specified, bipolar disorder, bulimia nervosa, or binge‐eating disorder. Additional exclusion criteria for all participants are reported in SM 1.1.

To account for potential developmental effects on DD,^35^ 80 participants were pseudo‐randomly selected from the greater HC sample to pairwise age‐match the acANs using a mathematical algorithm,^36^ that solves the assignment problem, that is, minimizes the sum of age differences within pairs. This resulted in a maximum difference of 0.5 years between the individuals within one pair.

### Clinical and demographic measures

BMI standard deviation scores (BMI‐SDS)^37^, ^38^ were computed to provide an age‐ and gender‐corrected index of weight‐to‐height ratio. A proxy‐measure of the socioeconomic status (SES) was assessed according to ISCO 08 (*ISCO – International Standard Classification of Occupations*);^39^ see SM 1.1 for details. Depressive symptoms were examined using the Beck Depression Inventory‐II (BDI‐II),^40^ and Eating‐disorder‐related psychopathology was assessed with the Eating Disorder Inventory (EDI‐2)^41^; see SM 1.2 for details. Internal consistency was acceptable (Cronbach's α = 0.799) for the complete EDI‐2 and excellent (Cronbach's α = 0.939) for BDI‐II. Numeric intelligence was estimated with the corresponding modules of the Wechsler Intelligence Scale for Children^42^ or the Wechsler Adult Intelligence Scale.^43^ Other pertinent information including, for example, clinical subtype and menstrual cycle was obtained using the SIAB‐EX^32^ supplemented with our own semi‐structured research interview.

### Experimental procedure

After consuming breakfast, participants completed a gamified DD task adapted from Scherbaum *et al*.^44^ between 9 a.m. and 10 a.m. using a laptop and a separate monitor placed in a standardized position.

**Fig. 1:**
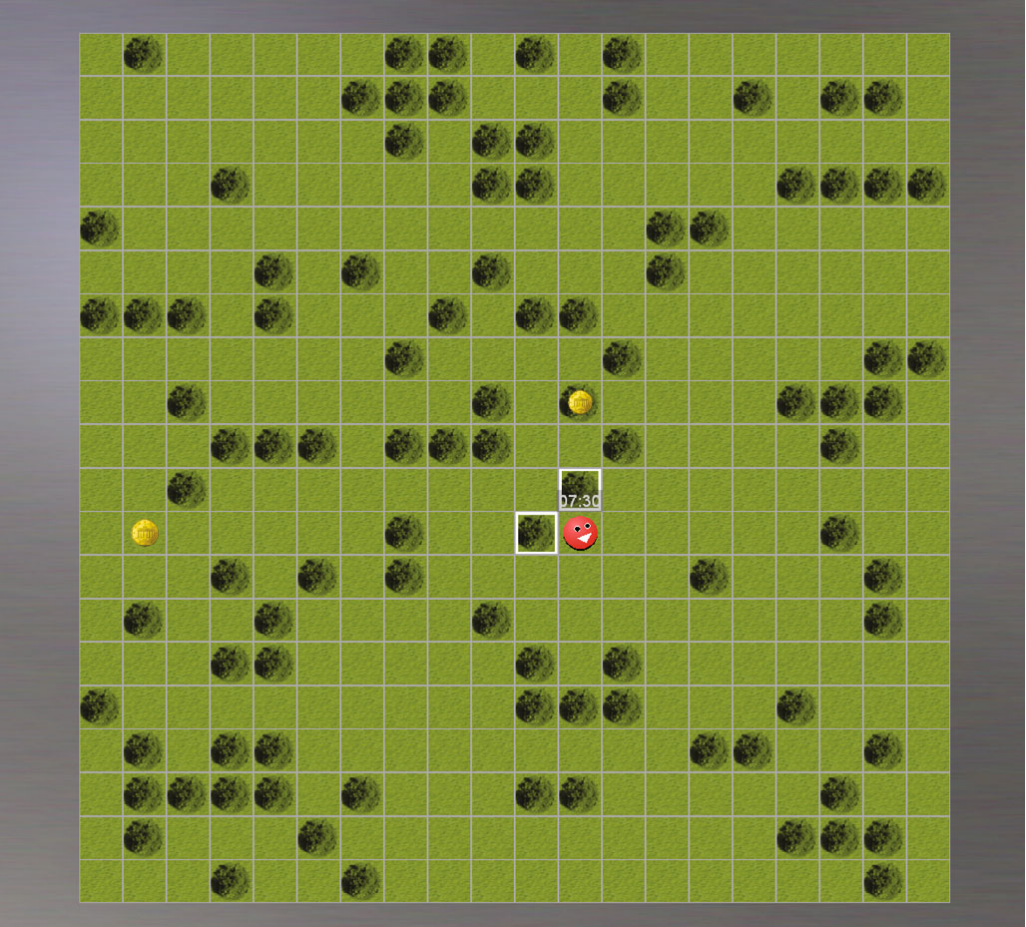
Design of the task. Trials were grouped into three blocks, each lasting 8 min. In each trial, participants could choose between a smaller, closer reward (SS) and a larger, more distant one (LL). The difference between the distances of the SS and the LL options could only be 1, 2, 4, or 7 squares. The choice was made by clicking with a computer mouse on one of the squares with a white contour adjacent to the red avatar. Unlike the task used by Scherbaum *et al*.,^44^ where each click only moved the avatar to an adjacent square, in this task the avatar moved autonomously after the first click to collect the reward positioned in the direction indicated by the first click, taking 1.3 s to move one square. The size of the coin was proportional to the reward value, with the smallest coin awarding 0.005€ and the largest 0.05€. Upon collecting a reward, participants were shown the total amount of monetary reward accumulated so far. There was no time limit for each trial, but since the remaining time was displayed above the avatar continuously, participants were incentivized to make quick decisions.

The task consisted of three blocks, each lasting 8 min. During each trial, participants controlled the first step of an avatar to collect one of two monetary rewards: a smaller, closer reward (SS) or a larger, more distant reward (LL, see Fig. 1). Once a direction toward one of the two rewards was selected, the avatar autonomously moved to collect it. Therefore, the closer reward was received sooner, and the more distant reward was received later. The participants had to wait until the avatar collected the chosen reward, with the waiting time being proportional to the distance between the avatar and the reward, before proceeding to the next trial. This requirement aimed to make the task experiential, thus increasing its ecological face validity relative to classical DD tasks previously employed in AN samples, which often relied on hypothetical choices.^23^, ^24^ In each trial, one option was objectively optimal, offering the highest reward per unit of time. Optimality was calculated as the reward magnitude divided by the time required for the avatar to reach it. Choosing the more distant reward, the opportunity costs increased: while the participant was waiting for the avatar to collect the chosen reward, they could not collect other rewards. On the contrary, since the overall time, but not the number of trials was fixed, collecting closer rewards resulted in more trials (i.e. more decisions to be taken) and allowed to collect a greater number of rewards. Each reward collected was added to a total loot, that together with the remaining time was shown continuously to the participants. Importantly, despite the fact that the LL option was more often the optimal one [odds‐ratio = 1.51 (0.03), meaning that in 60% of the trials the LL option was optimal], we confirmed that the overall proportion of trials in which the SS or LL option was optimal did not differ between the groups [odds‐ratio = 0.98 (0.03), *P* > 0.4]. On average, the difference in delay between the SS and LL options was mean (SD) = 4.5 (3.0)s, *P* < 0.001, and the difference in reward was mean (SD) = 5.0 (2.8)€cent, *P* < 0.001. To help participants familiarize themselves with the task contingencies (i.e. the distribution of delays and rewards for the SS and LL options), the main task was preceded by a visual introduction using screenshots, followed by a short tutorial. The precise instructions in German and an English translation are reported in the SM 1.3. The tutorial was statistically identical to the actual task but had a shorter duration (30 s). At the end of the tutorial, participants had the chance to ask questions, and if they required so, they could repeat the tutorial. However, this was never the case. All participants declared to have understood the task before the real task began.

Details regarding stimulus presentation, which followed the procedures of Scherbaum *et al*.,^44^ are reported in the SM 1.4. As reimbursement, participants received the credits earned in the experiment (mean [SD] = 4.80 [0.03]€, range = 3.82–5.29€ as a bonus to base compensation ≈€10/h).

### Data processing

To determine DD parameters, we followed Scherbaum *et al*.^44^ and excluded from the analysis trials with a reaction time <0.3s and exceeding 15s. However, in our sample no trial had to be excluded according to this criterion. For each participant and delay difference (LL‐SS), we determined an ‘indifference point’ as the ratio between the objective values of the SS and LL options at which the participant chose each option with equal frequency. This point reflects the subjective balance of value between the SS and LL reward, providing a basis to quantify individual discounting rates. We determined the individual discounting parameter k by fitting the indifference points to a hyperbolic function^45^ (SM 1.5).

### Statistical analysis

First, to directly test our hypothesis of reduced discounting in the AN group, we employed general linear models (GLMs) with the proportion of SS choices, area under the curve (AUC),^46^ and the log‐transformed discounting parameter *k* ^47^ as dependent variables, and age as a covariate, which is known to be an important determinant of DD.^48^

Second, to investigate whether potential group differences might reflect more/less rational behavior, we compared the proportion of optimal choices (i.e. having a higher rate of reward per unit of time) across groups. To this end, we employed binomial logistic regression models, with age as a covariate. We corrected for multiple comparisons using the False Discovery Rate (FDR) procedure.^49^

Hypothetically, group differences in the proportion of optimal choices might relate both to an ability to detect which option is optimal, or, in a scenario where the SS (or LL) option is more often the optimal choice, a bias toward the SS (or the LL) option. To gain a more nuanced understanding of whether the unexpectedly high proportion of SS choices in AN reflected a true preference for SS options, follow‐up analyses tested for group differences in the proportion of (i) LL choices when LL was the optimal choice, (ii) SS when SS was the optimal choice, and (iii) the discriminability (*d*′) and response bias (β) parameters from Signal Detection Theory (SDT).^50^ The discriminability (d′) captures the true ability to distinguish between optimal and suboptimal options, independent of any decision biases (SM 1.6). This provides a clearer measure of perceptual or cognitive ability. The response bias (β) reflects the tendency to favor a particular response, for example, the SS (SM 1.6). Importantly, trials where both options are equally optimal are informative but not required, as bias can be inferred from asymmetric error patterns across trials where different options are optimal. We used binomial logistic regression models and GLMs with age as a covariate to compare the proportions and SDT parameters between groups, respectively.

To investigate whether group findings were related to depressive symptoms or the severity of eating disorder symptoms, we conducted sensitivity analyses to relate the potential group findings in acAN to the explanatory clinical variables BMI‐SDS, BDI‐II, and EDI‐2 (SM 1.7). To investigate the potential effect of AN subtype, we compared AN‐r and AN‐bp separately to HC and to each other (SM 1.8). We also assessed the potential confounding effect of SES on discounting behavior and of IQ numeric on the task performance (SM 1.9).

## Results

### Sample characteristics

There were no significant differences between the groups regarding age, numeric intelligence, or SES. As expected, BMI and BMI‐SDS were lower while eating disorder and depressive symptoms were higher in the acAN group compared to HC (Table 1).

**Table 1:** Demographic and clinical characteristics of the sample.

	HC mean (SD)	acAN mean (SD)	*t*	*df*	*P*
Age	16.27 (2.51)	16.26 (2.48)	0.03	157.98	0.980
IQ numeric	11.56 (3.21)	10.70 (3.13)	1.08	30.81	0.287
BMI	20.89 (2.10)	14.66 (1.42)	22.00	138.78	<0.001
BMI‐SDS	0.05 (0.64)	−3.24 (1.07)	23.64	128.55	<0.001
EDI‐2 total	132.81 (25.35)	221.92 (44.18)	−15.04	113.48	<0.001
BDI‐II	4.57 (5.05)	26.29 (10.61)	−16.31	110.55	<0.001
SES	3.75 (0.92)	3.53 (0.83)	1.58	156.13	0.117

*Note*: All data of this table refer to the final sample considered in the analysis. acAN, Individuals with acute anorexia nervosa, HC, healthy control participants. Sixty‐five acAN individuals were of the restrictive subtype and 15 of the binge/purge subtype. All participants were female (sex assigned at birth) and all were of Caucasian ethnicity, except for one acAN (Asian) and one HC (other ethnicity). Group differences were tested using Welch's *t*‐tests.

Abbreviations: acAN, acute anorexia nervosa; BDI‐II, Beck Depression Inventory‐II; BMI, body mass index; BMI‐SDS, body mass index standard deviation score; EDI‐2, eating disorder inventory 2; IQ, intelligence quotient; SES, socioeconomic status.

### Discounting behavior

**Fig. 2:**
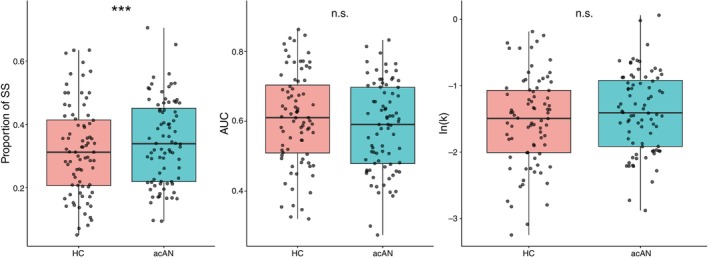
Group differences for the proportion of SS choices (left), AUC (center), and log‐transformed delay discounting parameter *k* (left) as computed with binomial logistic regression (proportion of SS) or a general linear model (AUC, k) with age as a covariate. No group differences for AUC or *k* were revealed (FDR‐corrected *q* > 0.2). However, in acAN, the odds of choosing the SS option were 13% higher (FDR‐corrected *q* < 0.0001, Table S1).

No group differences in DD (as measured using *k* or AUC) were detected, but the proportion of SS choices was higher in acAN compared to HC (Fig. 2, Table S1). Furthermore, this difference was more pronounced in patients with lower BMI‐SDS and more severe depressive and eating disorder symptoms (SM 2.1).

### Decision‐making optimality

**Fig. 3:**
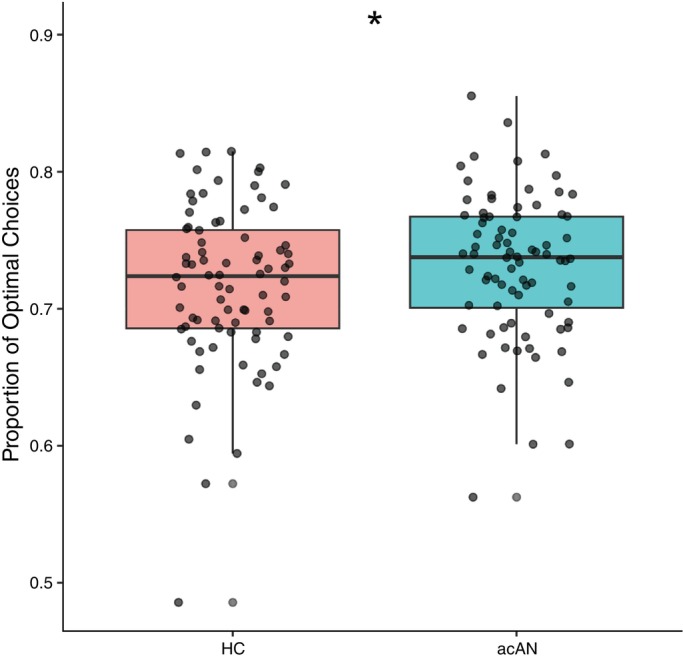
Group differences for the proportion of optimal choices as computed within a GLM with age as a covariate. In acAN, the odds of choosing the optimal option were 7% higher than HC, respectively (FDR‐corrected *q* = 0.04, Table S2).

**Fig. 4:**
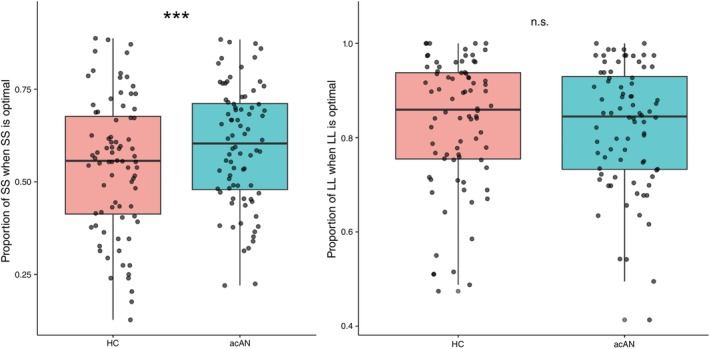
Group differences for the proportion of SS choices when this was optimal (left), and for the proportion of LL choices when this was optimal (right) as computed within a GLM with age as a covariate. While the proportion of SS choices when optimal was higher in acAN (FDR‐corrected *q* < 0.0001, odds 23% higher), no group differences were revealed for the proportion of LL choices (FDR‐corrected *q* > 0.2, Table S3).

The proportion of optimal choices was higher in acAN compared to HC (Fig. 3, Table S2). More precisely, our follow‐up analyses indicated that acAN made more SS choices when SS was the optimal choice compared to HC but did not make more LL choices when LL was the optimal choice (Fig. 4, Table S3). Furthermore, when the optimal choice was SS, the proportion of SS choices was higher in patients with more severe depressive symptoms and lower BMI‐SDS (SM 2.1, Figs S1,S2). However, no significant differences between AN‐r and AN‐bp were revealed (SM 2.2). Of note, choosing more often the SS option also caused the participants in the acAN group to perform more trials (Fig. S3), since the average duration of each trial was shorter, and no group difference in reaction times was revealed (*P* = 0.27, SM 2.3). The higher proportion of optimal choices did result in higher monetary gain on average in the AN group, but the difference was not significant (Fig. S3). Importantly, inclusion of SES as an additional covariate did not alter these results, and IQ numeric was not associated with task performance (SM 2.4).

### Signal detection theory

Supplementary SDT analysis did not reveal group differences in discriminability or response bias (Table S3).

## Discussion

In this study, we employed a novel gamified intertemporal choice task to investigate: (i) DD and (ii) decision‐making optimality in underweight participants with acute anorexia nervosa (acAN). Our task offered participants a direct experience of delay, enhancing its ecological face validity compared to traditional DD tasks. Specifically, rewards were positioned at distances corresponding to the time required for the avatar to collect them. Contrary to our research hypothesis, but consistent with previous studies in young patients with AN,^9^ even using this experiential approach we did not find shallower DD in AN: both the rate of DD (k) and the AUC did not differ between groups. However, the proportion of SS choices, as opposed to LL choices, was even increased in acAN in the current study. This unexpected choice behavior was not maladaptive in the context of our task. In fact, the acAN group made more optimal choices than the HC group. Optimality was defined by compensation based on total reward within a fixed time limit: The option yielding more money per unit time was optimal, the alternative suboptimal. Further examination revealed that the acAN group made more SS choices than HC when the SS option was optimal, but not when the LL option was optimal, suggesting that possibly acANs choose the SS option more often to improve their performance. To explore whether acAN exhibited a bias toward SS options or had a better ability to identify optimal choices, we applied SDT. This analysis did not support the notion that the acAN group's higher performance came from either being better at recognizing which option was optimal or from a general preference for SS options. Remarkably, however, choosing the SS option required more decisions throughout the task (acAN performed a higher number of trials) and therefore more decision‐making and physical effort. These findings suggest that, compared with the HC group, individuals with AN were either more motivated to achieve high performance and/or perceived effort as less costly, or even as rewarding.

Going beyond previous studies that relied mostly on hypothetical choices to compare discounting rates, we used a gamified task where participants experienced the actual waiting time involved in receiving a delayed reward.^51^, ^52^ Specifically, participants learned in real time that waiting for the expected reward prevented them from actively collecting other rewards. This type of design is most comparable with the DD paradigms used with non‐human animals^53^ and has been shown to not only enhance participant engagement but also increase the ecological validity of the results.^24^, ^26^ Moreover, a similar task setup proved effective in detecting steeper discounting in heroin addiction.^28^ However, even in this more realistic setting, we did not observe simple group differences in DD parameters. In this respect, our findings are similar to some,^16^, ^17^, ^20^, ^54^, ^55^, ^56^ but not all studies in AN,^9^, ^13^ and do not support our initial hypothesis that acAN might have a higher ability to delay gratification.

When considering the finding that the proportion of LL choices was lower in acAN compared to HC, it is important to note that seemingly impulsive choices can sometimes be advantageous. Generally, opportunity costs depend on environmental conditions and specifically on the average rate of reward from all other options.^57^ From an evolutionary perspective, securing a smaller, immediate reward at the right moment could enhance an individual's productivity and potentially increase their chances of survival.^58^ In such cases, a preference for SS options should not necessarily be interpreted as higher impulsivity. In our task, the introduction of a time limit enabled the identification of an optimal option, as it facilitated collecting more money per unit of time. This feature allowed to investigate whether differences in discounting behavior deviated from optimal decision‐making. Of note, using a similar task, Scherbaum *et al*.^28^ found that individuals with heroin addiction not only favored SS options, but also made less optimal choices compared to HC, which was interpreted as an indicator of higher impulsivity.^28^ In contrast, our study found that acAN participants showed a higher proportion of both SS and optimal choices. Specifically, the SS rate was higher in AN compared to HC when SS was the optimal choice, but not when LL was optimal. To further explore whether the higher SS choice rate reflected an inherent preference for SS options or a heightened ability to identify the optimal choice, we applied SDT,^50^ which quantifies these tendencies using two parameters: β (bias) and *d*′ (discriminability). No group differences in these parameters were observed. The absence of differences in the bias parameter (β) aligns with our results showing no differences in discounting parameters, suggesting that acAN participants did not have a stronger inherent preference for earlier rewards than HC. Similarly, the lack of differences in the discriminability parameter (*d*′) indicates that neither group demonstrated a superior ability to identify the optimal choice.

Importantly, however, the number of performed trials was higher in participants with AN due to their higher proportion of SS choices. A higher number of trials to be performed within the time limit presumably increased the attentional demand and therefore the effort that the participant had to dedicate to the task. Conversely, choosing the LL option allowed participants to relax for a longer period while waiting for the reward to be collected by the avatar. This finding aligns with results from a traditional DD study, where participants with AN exhibited reduced frontoparietal functional magnetic resonance imaging (fMRI) activation during decision‐making, alongside quicker and more consistent choice patterns, potentially indicating enhanced neural efficiency.^59^ Possibly reflecting higher efficiency in our task, choosing the SS option may not have felt particularly effortful for participants with AN. Similarly, studies that analyzed mouse cursor trajectories during a DD task^17^ and self‐control conflicts in daily life with ecological momentary assessment^60^ also indicated that individuals with AN may not require as much effortful inhibition compared to HC. Furthermore, our findings of more frequent SS choices in AN in the context of our task are generally in line with the recently proposed theory of learned industriousness in AN.^61^ According to this theory, excessive goal pursuit in the disorder might stem from a conditioned reward response to repeated high‐effort actions, which gradually makes effort exertion less aversive and even more rewarding, while not exerting effort might become associated to feelings of guilt and become negatively valenced. A recent study employing an effort discounting task found no clear behavioral support of higher effort willingness in AN, but fMRI findings suggested that effort investment was higher in patients during effort‐based decision‐making.^62^ However, neither King *et al*.^62^ nor the current study could test a central notion of the learned industriousness theory regarding the process of learning the value of effort, which would require an alternative experimental design.^63^ Another explanation is that, regardless of perceived effort, individuals with AN have perfectionistic tendencies aimed at maximizing performance^64^ and were found to be particularly vigilant to errors.^65^ Further support for this hypothesis, in addition to our results, comes from a bead‐sorting task study showing greater effort investment to achieve better performance in individuals with AN,^66^ and higher academic achievement observed in male and female students with AN.^7^

Of note, our study design can also not establish whether the higher proportion of SS choices in this task is an effect of undernutrition or is potentially a more persistent marker of risk for AN. While the detected group difference was more pronounced in patients with lower BMI‐SDS and more severe depressive and eating disorder symptoms, which is suggestive of the finding being state‐dependent, this interpretation remains speculative. Longitudinal data in former patients after weight restoration will be needed to confirm or refute this hypothesis.^67^ Furthermore, while previous reports indicated that DD might differ across AN subtypes,^9^, ^19^ we did not find any significant differences between subtypes in supplementary analyses.

Our findings should be interpreted with an awareness of certain important study limitations, along with those previously noted. First, our findings in a sample of mostly adolescent females with AN of the restrictive subtype and in the acutely underweight state may not generalize to older individuals (including males and diverse) with a longer duration of illness or a different AN subtype. Second, while the hypothesized preference for delayed rewards in acAN was not revealed in the present monetary task, results might differ in another context,^68^ that is, using a different task with exercise^69^ or food rewards,^18^ or delays longer than those used here (up to ~20s). Furthermore, while larger than most DD studies in psychiatry,^9^ our sample had limited power to detect small effects in *k* or AUC. Third, since we did not collect subjective ratings on the perceived effort and brief strategy reports, we cannot disentangle partially overlapping explanations involving effort evaluation and increased motivation to perform better. Future studies should therefore complement behavioral measures with such information to better capture the motivational processes underlying task performance, or employ neurophysiological measures to reveal distinct activation patterns corresponding to these processes, engaging reward/approach‐related, punishment/avoidance‐related, or effort and cognitive control brain regions or networks.^62^ Finally, the between‐group difference in the average number of completed trials was small (about four trials in total across the three blocks) indicating only a modest disparity in executed effort. Accordingly, inferences about effort‐related mechanisms should be made with caution. Similarly, the amount of monetary compensation won was numerically higher in AN but did not differ significantly between groups. Therefore, these differences are unlikely to be a primary driver of the marked differences in eating and exercise behavior commonly observed in AN.^69^

To conclude, even with an experiential and more ecologically valid task, we did not reveal the hypothesized reduction in DD in AN. We speculate that the observed higher proportion of optimal SS choices in the context of the task might be driven by a different evaluation of effort in individuals with AN. The characteristics observed in our sample of adolescent and young females appear advantageous in specific contexts, such as those simulated by our experiential task. Particularly in patients that are motivated toward recovery, these characteristics might be strategically leveraged to inform the development of new and enhanced therapeutic approaches in the future.^70^ Specifically, therapeutic approaches could draw on the patient's capacity for sustained effort by reframing recovery‐oriented behaviors as structured, goal‐driven tasks (e.g. collaboratively setting specific eating goals, monitoring progress, and providing clear feedback on achievement). Such strategies aim not to simplify the challenge of overcoming restrictive drives but to harness an existing cognitive style for adaptive purposes.

## Author contributions

Conceptualization was performed by S.S., K.G., K.W., V.R., and S.E. Methodology was developed by F.B., S.E., M.S., and S.S. Software was provided by F.B., M.S., and S.S. Formal Analysis was performed by F.B., and S.S. Investigation was done by F.B., J.K., M.S., and N.K. Resources were provided by K.G., K.W., V.R., and S.E. Data Curation was performed by F.B. Original Draft was written by F.B., and S.E. Review and Editing were performed by F.B., J.K., M.S., M.S., N.K., K.G., K.W., V.R., S.S., and S.E. Visualization was performed by F.B., and S.S. Supervision was performed by J.K., S.S., V.R., and S.E. Project Administration was done by S.E. Funding Acquisition was done by S.E.

## Funding statement

This work was supported by the Deutsche Forschungsgemeinschaft (EH 367/5‐1 and SFB 940/2). The authors gratefully acknowledge the computing time provided by them on the high‐performance computers at the NHR Center NHR@TUD. This is funded by the Federal Ministry of Education and Research and the state governments participating on the basis of the resolutions of the GWK for the national high‐performance computing at universities (http://www.nhr-verein.de/unsere-partner).

## Disclosure statement

VR has received payment for consulting and writing activities from Lilly, Novartis, and Shire Pharmaceuticals/Takeda, lecture honoraria from Lilly, Novartis, Shire Pharmaceuticals/Takeda, and Medice Pharma, and support for research from Shire/Takeda and Novartis. VR has carried out clinical trials in cooperation with Novartis, Shire Pharmaceuticals/Takeda, and Otsuka companies. VR has no financial relationship with the organizations that sponsored the research. The other authors declare no conflict of interest.

## Supporting information


**Supplementary Material S1.** 1 Methods.
1.1 Sample description.
1.2 Clinical and Demographic Measurements.
1.3 Task instructions.
1.4 Stimulus presentation.
1.5 Hyperbolic discounting of the delay discounting parameter.
1.6 Signal detection theory.
1.7 Relationships between clinical variables and findings.
1.8 Relationships between AN‐subtype and findings.
1.9 Analysis of potential confounding effect of SES and IQ numeric.
2. Methods.
2.1 Relationships between clinical variables and findings.
2.2 Relationships between AN‐subtype and findings.
2.3 Group comparison of reaction times.
2.4 Effects of SES and IQ numeric.
**Figure S1.** Significant associations between proportion of smaller‐sooner (SS) choices and clinical characteristics for participants in the acute anorexia nervosa (acAN) group: body mass index‐standard deviation score (BMI‐SDS) (left, *t*[78] = −5.53, *P* < 0.0001), BDI‐II (center, *t*[76] = 4.01, *P* = 0.0001), and EDI‐2 (right, *t*[71] = 2.38, *P* = 0.017).
**Figure S2.** Significant associations between proportion of smaller‐sooner (SS) choices when SS was the optimal choice and clinical characteristics for participants in the acute anorexia nervosa (acAN) group: BMI‐SDS (left, *t*[78] = −3.85, *P* < 0.0001), BDI‐II (right, *t*[76] = 2.59, *P* = 0.009).
**Figure S3.** Group effects on number of trials completed (left), and total points won (right). For each 20 points, 0.01€ was paid to the participants. Participants performed on average 143 trials, and acute anorexia nervosa (acAN) performed four more than healthy controls (HC) (*P* = 0.03). For total points won, no group differences were revealed (*P* = 0.156).
**Table S1.** Group differences in discounting behavior.
**Table S2.** Group differences in optimality.
**Table S3.** Group differences in proportion of SS/LL choices when they were optimal, discriminability and response bias.

## Data Availability

The data that support the findings of this study are available from the corresponding author upon reasonable request.
