# Supplementary material for: Intact delay discounting but more optimal reward‐based decisions in anorexia nervosa during an experiential task
Source: Psychiatry Clin Neurosci. 2026 Feb 18;80(5):409–16. doi: 10.1111/pcn.70041 (PMC13139826; doi:10.1111/pcn.70041)
Supplement: Supplementary file 1 — Supplementary Material S1. 1 Methods. 1.1 Sample description. 1.2 Clinical and Demographic Measurements. 1.3 Task instructions. 1.4 Stimulus presentation. 1.5 Hyperbolic discounting of the delay discounting parameter. 1.6 Signal detection theory. 1.7 Relationships between clinical variables and findings. 1.8 Relationships between AN‐subtype and findings. 1.9 Analysis of potential confounding effect of SES and IQ numeric. 2. Methods. 2.1 Relationships between clinical variables and findings. 2.2 Relationships between AN‐subtype and findings. 2.3 Group comparison of reaction times. 2.4 Effects of SES and IQ numeric. Figure S1. Significant associations between proportion of smaller‐sooner (SS) choices and clinical characteristics for participants in the acute anorexia nervosa (acAN) group: body mass index‐standard deviation score (BMI‐SDS) (left, t[78] = −5.53, P < 0.0001), BDI‐II (center, t[76] = 4.01, P = 0.0001), and EDI‐2 (right, t[71] = 2.38, P = 0.017). Figure S2. Significant associations between proportion of smaller‐sooner (SS) choices when SS was the optimal choice and clinical characteristics for participants in the acute anorexia nervosa (acAN) group: BMI‐SDS (left, t[78] = −3.85, P < 0.0001), BDI‐II (right, t[76] = 2.59, P = 0.009). Figure S3. Group effects on number of trials completed (left), and total points won (right). For each 20 points, 0.01€ was paid to the participants. Participants performed on average 143 trials, and acute anorexia nervosa (acAN) performed four more than healthy controls (HC) (P = 0.03). For total points won, no group differences were revealed (P = 0.156). Table S1. Group differences in discounting behavior. Table S2. Group differences in optimality. Table S3. Group differences in proportion of SS/LL choices when they were optimal, discriminability and response bias. [file PCN-80-409-s001.docx]

**Intact Delay Discounting but More Optimal Reward-Based Decisions in Anorexia Nervosa during an Experiential Task**

***Supplementary Material***

1. **Methods**

*1.1 Sample description*

Participants of both groups were excluded if they reported psychotropic drug intake within the last 4 weeks, being pregnant or breast feeding, clinically relevant anemia, being younger than 12 or older than 30, having an IQ below 85, suffering from organic brain syndrome, dementia, schizophrenia, bipolar disorder, drug abuse, obesity (BMI>94th percentile for age<18, BMI>28 for age 18 and older) as well as chronic medical or neurological illnesses that could affect appetite, eating behavior or body weight (e.g. diabetes).

Healthy control participants (HC), were recruited either via advertisement among high school and university students or had participated in previous studies. HC were excluded if they had a lifetime diagnosis of any psychiatric disorder, the lowest lifetime BMI below the 10th percentile (below age 18) or <17.5 (for age 18 and older) or if they were currently underweight, or showed abnormal eating behavior (diet, binge eating). Additional exclusion criteria for all participants included intelligence quotient (IQ)<85; psychotropic medication within four weeks prior to the study (except selective serotonin reuptake inhibitors; n=2 acAN); current substance abuse; current inflammatory, neurologic or metabolic illness; chronic medical or neurological illness that could affect appetite, eating behavior or body weight; clinically relevant anemia; pregnancy; breast feeding.

Active psychiatric comorbidity was diagnosed in acAN by an expert clinician after careful chart review and consultation with clinical staff. Other pertinent information was collected from all participants using our own semi-structured research interview which includes several specific questions to assess menstruation history, weight history, general medical and pharmaceutical history, family psychiatric history, smoking status, and socioeconomic factors (e.g., educational level, occupation, family status, current living situation). Socioeconomic status (SES) was measured following the ISCO guidelines (https://www.ilo.org/wcmsp5/groups/public/---dgreports/---dcomm/---publ/documents/publication/wcms_172572.pdf). Briefly, SES was assessed for the participant and their parents or other persons sharing the same household according to the educational level/occupation group [range: 0 (lowest), leaving school without graduation – 5 (highest), graduating from university]. For study participants who still lived with their parents or guardians, the highest SES in the family/household was used (Patrick et al., 2004).

Study data were managed using the Research Electronic Data Capture (https://www.project-redcap.org/) web interface.

*1.2 Clinical and Demographic Measurements*

Eating disorder-related psychopathology was assessed with the German short version of the Eating Disorder Inventory (EDI-2 (Paul & Thiel, 2005)), a reliable and well-validated (Kappel et al., 2012; Thiel & Paul, 2006) self-report instrument with 64 items on eight subscales (e.g., drive for thinness, body dissatisfaction, bulimia, perfectionism, and a global score) rated on a scale ranging from 1 (never) to 6 (always) reﬂecting symptom frequency. Internal consistency of the complete EDI-2 was acceptable (Cronbach’s α=.799). Depressive symptoms were examined using the German version of the Beck Depression Inventory–II (BDI-II; (Hautzinger et al., 2009)), a self-report instrument with 21 items rated on a 4-point scale ranging from 0 (not at all) to 3 (extreme) reﬂecting their presence and intensity in the last two weeks. Higher total scores indicate more severe symptoms. The reliability and discriminant validity of the BDI-II is well-established (Kühner et al., 2007). Internal consistency was excellent (Cronbach’s α=.939).

*1.3 Task instructions*

Participants received the following instructions in German, which they were able to read at their own pace using a mouse button to move on (English translation follows):

1. Willkommen und vielen Dank, dass du an diesem Experiment teilnimmst! Im Folgenden erkläre ich dir kurz, was deine Aufgabe im Experiment sein wird. Du wirst dabei alle möglichen Situationen, die auftreten können, kennenlernen. Du erhähltst die Möglichkeit, alles selbst auszuprobieren.
2. Bitte lies die folgenden Erklärungen aufmerksam durch. Wenn du Fragen hast, stelle diese bitte am Ende der Instruktion.
3. Du wirst dich im Experiment mit deiner Spielfigur frei durch eine virtuelle Welt bewegen können. Deine Aufgabe ist es, in dieser Welt in begrenzter Zeit (3 Blöcke a 8 Minuten) Schätze zu sammeln, die immer wieder auftauchen.
4. Das Tolle für dich ist: die Summe der gesammelten Schätze wird dir am Ende als Bonus ausbezahlt!
5. Ich zeige dir nun die Welt und erkläre alles etwas genauer.
6. Dies ist das Spielfeld, eine kleine Welt, in der du dich frei bewegen kannst.
7. Das ist deine Spielfigur, mit der du dich in der virtuellen Welt bewegst.
8. In der Welt werden immer wieder Schätze auftauchen.
9. Dein Ziel ist es, Schätze einzusammeln. Dazu musst du, ein direkt an deine Figur angrenzendes Feld in Richtung des jeweiligen Schatzes anklicken.
10. Deine Figur wird sich dann automatisch in Richtung des Schatzes bewegen.
11. Je ferner der Schatz, desto mehr Zeit wird die Figur brauchen um es zu schaffen.
12. Die Figur kann sowohl auf Felder mit Gras, als auch auf Felder mit Wald ziehen. Das macht keinen Unterschied.
13. Für jeden gesammelten Schatz erhälst du einen Betrag, der durch die Größe der Münze auf dem Feld des Schatzes angezeigt wird.
14. Die größte Münze bedeutet 5 Cent.
15. Die kleinste Münze bedeutet 0,5 Cent.
16. Aber Achtung: wenn zwei Schätze gleichzeitig zur Wahl stehen, dann musst du dich für einen der beiden entscheiden.
17. Der jeweils andere Schatz wird dann verschwinden.
18. Du kannst also den nahen, kleinen Schatz wählen.
19. Oder den weiteren, aber größeren Schatz.
20. Über deiner Figur wird dir angezeigt, wie viel Zeit du für diesen Block noch zur Verfügung hast.
21. Unter deiner Figur wird dir nach Einsammeln eines Schatzes angezeigt, welchen Betrag du bereits insgesamt gesammelt hast.
22. Es gibt insgesamt 3 Blöcke, zwischen denen eine kurze Pause ist.
23. Jetzt drück die linke Maustaste um fortzusetzen oder die rechte Maustaste um diese Instruktionen noch einmal zu lesen.
24. Du kannst nun selbst zum Test kurz die Welt betreten.
25. Du hast eine halbe Minute Zeit, um die Welt auszuprobieren und Schätze zu sammeln.
26. Versuche auf die Felder direkt neben der Figur zu klicken, um deine Figur zu einem Schatz zu senden, und mach dich mit der Steuerung vertraut.
27. Die Schätze, welche du jetzt sammelst, spielen für das Experiment noch keine Rolle. Du kannst es also ganz entspannt angehen!
28. Du hast nun die Welt erfolgreich ausprobiert! Solltest du noch Fragen haben, wende dich bitte nun an den Versuchsleiter!

These instructions can be translated to:

1. Welcome and thank you very much for taking part in this experiment! In the following, I will briefly explain what your task in the experiment will be. You will get to know all possible situations that may occur, and you will have the opportunity to try everything out yourself.
2. Please read the following explanations carefully. If you have any questions, please ask them at the end of the instructions.
3. In the experiment, you will be able to move freely through a virtual world with your game avatar. Your task is to collect treasures in this world within a limited amount of time (3 blocks of 8 minutes each), which will keep reappearing.
4. The great thing for you is: the total amount of the collected treasures will be paid out to you as a bonus at the end!
5. I will now show you the world and explain everything in more detail.
6. This is the playing field, a small world in which you can move freely.
7. This is your game avatar, with which you move around in the virtual world.
8. Treasures will keep appearing in the world.
9. Your goal is to collect treasures. To do this, you must click on a field directly adjacent to your avatar in the direction of the respective treasure.
10. Your avatar will then automatically move toward the treasure.
11. The farther away the treasure is, the more time the avatar will need to reach it.
12. The avatar can move on fields with grass as well as on fields with forest. That makes no difference.
13. For each treasure collected, you will receive an amount that is indicated by the size of the coin on the treasure’s field.
14. The largest coin means 5 cents.
15. The smallest coin means 0.5 cents.
16. But be careful: if two treasures appear at the same time, you have to decide between the two.
17. The other treasure will then disappear.
18. So, you can choose the nearby small treasure.
19. Or the more distant but larger treasure.
20. Above your avatar, you will see how much time you still have available for this block.
21. Below your avatar, after collecting a treasure, you will see the total amount you have collected so far.
22. There are a total of 3 blocks, with a short break in between.
23. Now press the left mouse button to continue, or the right mouse button to read these instructions again.
24. You can now enter the world yourself for a short test.
25. You have half a minute to try out the world and collect treasures.
26. Try clicking on the fields directly next to the avatar to send your avatar toward a treasure, and get familiar with the controls.
27. The treasures you collect now are not relevant for the actual experiment. You can therefore take it easy!
28. You have now successfully tried out the world! If you still have any questions, please contact the experimenter now!

*1.4 Stimulus presentation*

Stimuli were presented on a grey background on a 19-in. screen running at a resolution of 1,280 × 1,024 pixels (60-Hz refresh frequency). Participants performed their responses with a standard computer mouse (Fujitsu M530, Laser USB). The task was implemented in Matlab R2014a, using the Psychophysics Toolbox 3 extension (Brainard, 1997).

*1.5 Hyperbolic discounting of the delay discounting parameter*

The subjective value V of any monetary amount A in the number of steps necessary for its collection S was modeled hyperbolically (Green et al., 1994), and the degree of this devaluation was described by the discounting parameter $k$:

$$V=\frac{A}{1+k*S}$$

High k values reflect steep discounting of delayed outcomes and, therefore, a tendency to favor closer or more immediate options.

*1.6 Signal detection theory*

Signal Detection Theory (SDT) is a framework used to measure the ability to differentiate between signal (target) and noise (non-target) in decision-making tasks. It quantifies two key parameters: sensitivity (d’) and response bias (β). In the context of our task, we define the hit rate as the proportion (Green et al., 1994) of larger-distant (LL) choices when LL is the optimal option, and the correct rejection rate as the proportion of smaller-closer (SS) choices when SS is the optimal option. Similarly, we define the false alarm rate as the proportion of LL choices when SS is optimal, and the miss rate as the proportion of SS choices when LL is the optimal option.

Based on these definitions, the sensitivity (d’) can be calculated using the formula:

$$d^{'}=Z\left( hit rate \right)-Z(false alarm rate)$$

where $Z$ is the z-score transformation that converts a proportion to a value on the standard normal distribution. The z-score for a proportion $p$ can be calculated as:

$$Z\left( p \right)=\phi^{-1}(p)$$

where $\phi^{-1}$is the inverse of the cumulative distribution function (CDF) of the standard normal distribution.

The response bias (β) can be calculated using the formula:

$\beta=\text{exp}\left( \frac{{Z(false alarm rate)}^{2}-{Z(hit rate)}^{2}}{2} \right)$.

*1.7 Relationships between clinical variables and findings*

To investigate whether group findings were related to depressive symptoms or the severity of eating disorder symptoms, we used GLMs with (i) the proportion of SS choices, as well as (ii) the proportion of optimal choices, and of (iii) SS choices when SS was the optimal choice as dependent variables. In each model, we tested the effect of BDI-II, BMI-SDS, or EDI-2 as covariates of interest, while accounting for the effects of age as a confounding variable.

- 1. *Relationships between AN-subtype and findings*

To investigate the potential effect of AN-subtype, we employed GLMs similar to our main analyses (for proportion of SS, optimal, SS/LL when optimal; k, AUC, number of valid trials, total amount won) where participants in the AN group were assigned to their AN subtype instead (AN-r or AN-bp). We subsequently computed post-hoc t-tests: (i) AN-r vs. HC, (ii) AN-bp vs. HC, and (iii) AN-r vs. AN-bp. We corrected for multiple comparisons using the False Discovery Rate (FDR) procedure as in the main analyses.

- 1. *Analysis of potential confounding effect of SES and IQ numeric*

While no group differences between SES and IQ numeric were revealed, we assessed the potential confounding effects of these variables on discounting behavior (SES) and performance (IQ numeric). Since socioeconomic background may influence how rewards are perceived, with the same nominal reward potentially being valued differently across SES levels, we considered supplemental models for the proportion of SS and optimal choices and for the discounting parameters in which SES was added as a covariate. Similarly, we assessed the effect of IQ numeric on performance (total amount win) by including it as an additional covariate.

1. **Methods**

*2.1 Relationships between clinical variables and findings*

The proportion of SS choices was increased in patients with lower BMI-SDS (t(78)=-5.53, p<.0001), more severe depressive symptoms (t(76)=4.01, p=.0001), and more severe eating disorder symptoms (t(71)=2.38, p=.017), Figure S1. We did not detect any significant relationship between the proportion of optimal choices and clinical variables. However, when the optimal choice was SS, the proportion of SS choices was increased in patients with lower BMI-SDS(t(78)=-3.85, p=.0001), and more severe depressive symptoms (t(76)=2.59, p=.009), Figure S2.

*2.2 Relationships between AN-subtype and findings*

For the AN-r subtype, the results were statistically identical as in the whole AN group. In particular, compared to HC, in AN-r, the proportion of SS (p<.001) and rational choices (p~.01) was higher, as well as the number of valid trials (p~.04). In the AN-bp subtype, the proportion of SS choices was higher than in the HC group (p~.04), but not the proportion of rational choices (p~.66), the proportion of SS rational choices (p~.08), and the number of trials (p~.22). Just as for the whole AN group, no differences between AN-r or AN-bp and HC was revealed for the other parameters (AUC, k, amount win, proportion of LL rational, discriminability and response bias, p>.15). Importantly, no difference in the above parameters was significant when comparing AN-r with AN-bp directly (p>.25). Therefore, the lack of findings in the AN-bp group might also be due to the reduced statistical power in this small subsample (n=15).

*2.3 Group comparison of reaction times*

To rule out the possibility that the increased number of trials in the AN group was related to faster reaction times, we employed an LME with log-transformed reaction times as dependent variable, random intercept, group as predictor of interest and age as possible confounding variable. No significant group effect was revealed (t=1.11, p=.27). The mean (SD) reaction time for the AN group was: 1.39(0.43)s, for the HC group was: 1.43(0.44)s.

*2.4 Effects of SES and IQ numeric*

There was no association between SES and the proportion of optimal choices or discounting parameters (p>.070), but participants with higher SES had a higher proportion of SS choices (p=.036). However, inclusion of SES as an additional covariate did not alter our results from the main analyses (no group differences for discounting parameters k and AUC, but increased proportion of SS and optimal choices in the acAN group). To assess whether IQ numeric was associated with task performance we fitted a GLM with amount win as dependent variable and group, age, and IQ numeric as predictors. While there was a significant effect of age (p=.002), the effect of IQ numeric was not significant (p=.10) and the group difference was not significant (p=.98) as in the main analysis without IQ numeric.

**Figures**


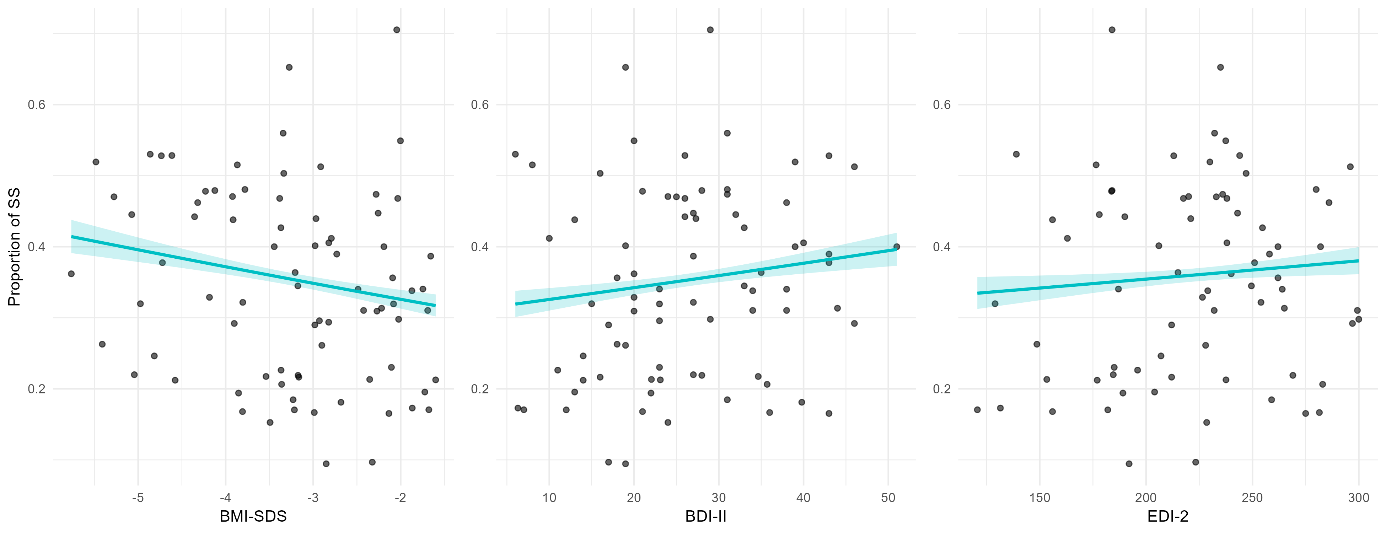


**Figure S1**: Significant associations between proportion of SS choices and clinical characteristics for participants in the acAN group: BMI-SDS (left, t(78)=-5.53, p<.0001), BDI-II (center, t(76)=4.01, p=.0001), and EDI-2 (right, t(71)=2.38, p=.017).


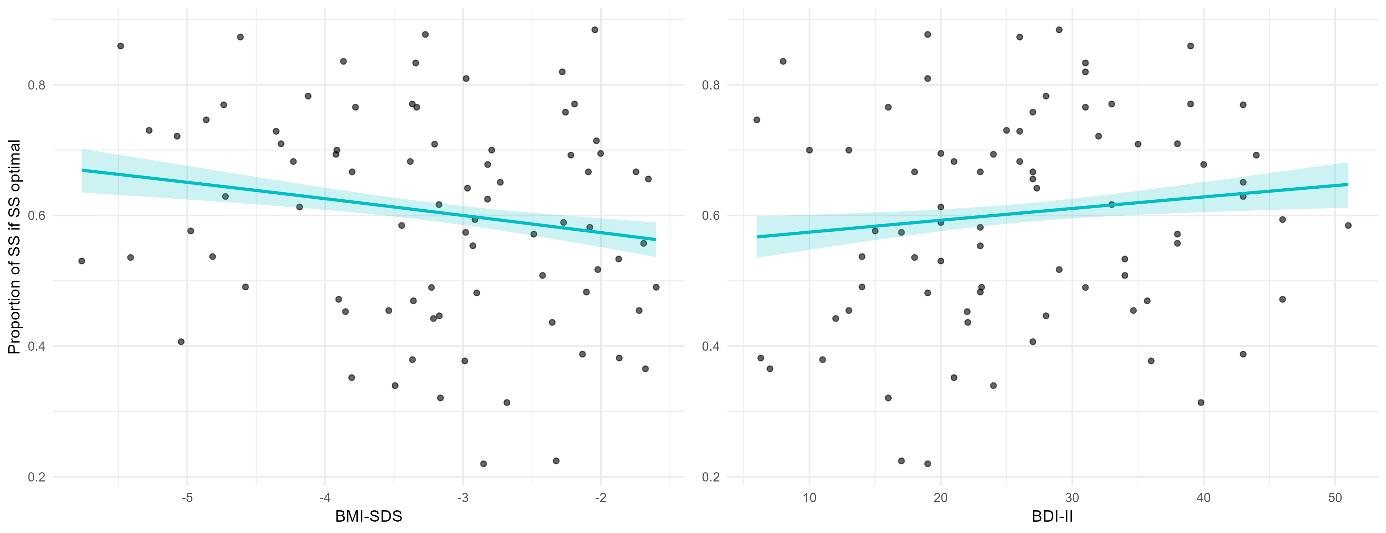


**Figure S2**: Significant associations between proportion of SS choices when SS was the optimal choice and clinical characteristics for participants in the acAN group: BMI-SDS (left, t(78)=-3.85, p<.0001), BDI-II (right, t(76)=2.59, p=.009).

**
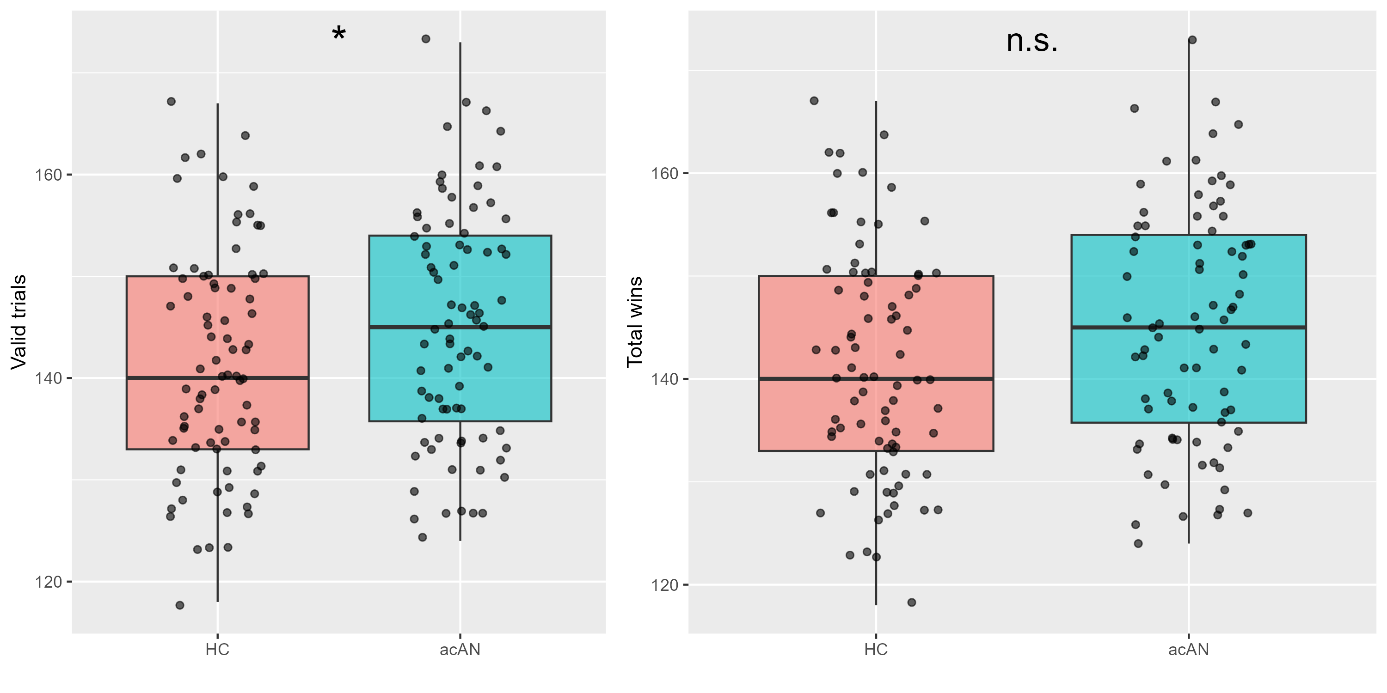
**

**Figure S3**: Group effects on number of trials completed (left), and total points won (right). For each 20 points, 0.01€ was paid to the participants. Participants performed on average 143 trials, and acAN performed 4 more than HC (p=.03). For total points won, no group differences were revealed (p=0.156).

**Tables**

**Table S1. Group differences in discounting behavior.**

| Outcome | **Intercept** | **Group (acAN)** | **Age** |
| --- | --- | --- | --- |
| **AUC** | 0.61(0.01) t=41.54 p=<.0001 | -0.03(0.02) t=-1.22 p=.2252 q=.2252 | -0.00(0.01) t=-.364 p=.7159 |
| **log(k)** | 1.53(0.07) t=-21.29 p=<.0001 | 0.13(0.10) t=1.31 p=.1911 q=.2252 | 0.01(0.05) t=.27 p=.7862 |
| **Proportion of SS** | 0.49(0.01) z=35.65 p<.0001 | 1.13(0.03) z=4.29 p<.0001 q<.0001 | 1.00(0.01) z=-0.22 p=.8297 |

Group differences for AUC and log(k) were computed within a general linear model with age as covariate, while for proportion of SS choices binomial logistic regression was used. For all effects we report the model estimate with standard error, statistics (t-values for AUC, and Wald z-statistic for proportions), and p-values. For proportions, the model estimates represent odds-ratios. We applied an FDR correction (FDR<.05) across all outcomes (including proportion of optimal choices, see Table 3) for group effects and also report corrected p-values (q).

**Table S2. Group differences in optimality.**

| Outcome | **Intercept** | **Group (acAN)** | **Age** |
| --- | --- | --- | --- |
| **Proportion of optimal choices** | 2.53(0.05) z=44.46 p<.0001 | 1.07(0.03) z=2.29 p=.0218 q=.0436 | 1.04(0.02) z=2.52 p=0.0118 |

Group differences for the proportion of optimal choices as estimated using binomial logistic regression with age as a covariate. We report the model estimate with standard error, Wald z-statistic, and p-values. The model estimate for the intercept represents an odds-ratio. We applied an FDR correction (FDR<.05) across all outcomes (including those related to discounting, see Table 2) for group effects and also report the corrected p-values (q).

**Table S3. Group differences in proportion of SS/LL choices when they were optimal, discriminability and response bias.**

| Outcome | **Intercept** | **Group (acAN)** | **Age** |
| --- | --- | --- | --- |
| **Proportion of SS when optimal** | 1.26(0.04) z=7.62 p<.0001 | 1.23(0.05) z=4.81 p<.0001 q<.0001 | 1.04(0.02) z=1.68 p=.0931 |
| **Proportion of LL when optimal** | 4.64(0.15) z=48.32 p<.0001 | 0.94(0.04) z=-1.32 p=.1868 q=.2166 | 1.04(0.02) z=1.93 p=.0539 |
| **Discriminability** | 1.24(0.04) t=29.26 p<.0001 | 0.08(0.06) t=1.26 p=.2085 q=.2166 | 0.03(0.03) t=1.15 p=.2517 |
| **Response bias** | 0.50(0.06) t=8.76 p<.0001 | -0.10(0.08) t=-1.24 p=.2166 q=.2166 | -0.01(0.04) t=-0.14 p=.8861 |

Group differences for discriminability and response bias were computed within a general linear model with age as covariate, while for proportions binomial logistic regression was used. For all effects we report the model estimate with standard error, statistics (t-values for AUC, and Wald z-statistic for proportions), and p-values. For proportions, the model estimates represent odds-ratios. We applied an FDR correction (FDR<.05) across all outcomes for group effects and for group effects we also report corrected p-values (q). A discriminability value greater than 1 suggests that the participant can reliably distinguish when SS is the optimal choice versus when LL is the optimal choice, performing well above chance. Here, a Response bias greater than 0.5 would indicate an a priori inclination to opt for the SS option.

**References**

Bernardoni, F., Bernhardt, N., Pooseh, S., King, J. A., Geisler, D., Ritschel, F., Boehm, I., Seidel, M., Roessner, V., Smolka, M. N., & Ehrlich, S. (2020). Metabolic state and value-based decision-making in acute and recovered female patients with anorexia nervosa. *Journal of Psychiatry & Neuroscience: JPN*, *45*(3), 190031. https://doi.org/10.1503/jpn.190031

Bernardoni, F., Geisler, D., King, J. A., Javadi, A.-H., Ritschel, F., Murr, J., Reiter, A. M. F., Rössner, V., Smolka, M. N., Kiebel, S., & Ehrlich, S. (2017). Altered Medial Frontal Feedback Learning Signals in Anorexia Nervosa. *Biological Psychiatry*. https://doi.org/10.1016/j.biopsych.2017.07.024

Brainard, D. H. (1997). The Psychophysics Toolbox. *Spatial Vision*, *10*(4), 433–436. https://doi.org/10.1163/156856897X00357

Green, L., Fry, A. F., & Myerson, J. (1994). Discounting of Delayed Rewards: A Life-Span Comparison. *Psychological Science*, *5*(1), 33–36. https://doi.org/10.1111/j.1467-9280.1994.tb00610.x

Hautzinger, M., Keller, F., Beck, A. T., & Kühner, C. (2009). *Beck Depressions-Inventar: BDI II ; Manual*. Pearson Assessment.

Kappel, V., Thiel, A., Holzhausen, M., Jaite, C., Schneider, N., Pfeiffer, E., Lehmkuhl, U., & Salbach-Andrae, H. (2012). Eating Disorder Inventory-2 (EDI-2): Normierung an einer Stichprobe normalgewichtiger Schüler im Alter von 10 bis 20 Jahren und an Patientinnen mit Anorexia nervosa. *Diagnostica*, *58*(3), 127–144. https://doi.org/10.1026/0012-1924/a000069

Kühner, C., Bürger, C., Keller, F., & Hautzinger, M. (2007). [Reliability and validity of the Revised Beck Depression Inventory (BDI-II). Results from German samples]. *Der Nervenarzt*, *78*(6), 651–656. https://doi.org/10.1007/s00115-006-2098-7

Patrick, K., Norman, G. J., Calfas, K. J., Sallis, J. F., Zabinski, M. F., Rupp, J., & Cella, J. (2004). Diet, Physical Activity, and Sedentary Behaviors as Risk Factors for Overweight in Adolescence. *Archives of Pediatrics & Adolescent Medicine*, *158*(4), 385. https://doi.org/10.1001/archpedi.158.4.385

Paul, T., & Thiel, A. (2005). *Eating Disorder Inventory-2 (EDI-2): Deutsche Version*. Hogrefe.

Thiel, A., & Paul, T. (2006). Test-retest reliability of the Eating Disorder Inventory 2. *Journal of Psychosomatic Research*, *61*(4), 567–569. https://doi.org/10.1016/j.jpsychores.2006.02.015
